# Compartmentalization of TNF and IL-6 in meningitis and septic shock

**DOI:** 10.1155/S096293519300002X

**Published:** 1993

**Authors:** Anders Waage, Alfred Halstensen, Terje Espevik, Petter Brandtzæg

**Affiliations:** 1 Institute of Cancer Research University of Trondheim Norway; 2 Section of Haematology University of Trondheim Norway; 3 Department of Internal Medicine Haukeland University Hospital Bergen Norway; 4 Department of Pediatrics Ullevål University Hospital Oslo Norway

## Abstract

We examined the compartmentalization of bioactive tumour necrosis factor (TNF) and interleukin 6 (IL-6) to the subarachnoid space and systemic circulation in patients with meningococcal meningitis and septic shock/bacteraemia. In patients with meningitis, median levels of TNF in 31 paired samples of cerebrospinal fluid (CSF) and serum were respectively 783 pg/ml and below detection limit (p < 0.001) and median levels of IL-6 were 150 ng/ml and 0.3 ng/ml (p < 0.0001). In patients with septic shock without meningitis, median levels in paired samples of CSF and serum were respectively below detection limit and 65 pg/ml (not significant, (ns)) (TNF, eleven patients) and 1.3 ng/ml–3 ng/ml (ns) (IL-6, nine patients). The data show that TNF and IL-6 are localized to the subarachnoid space in patients with meningitis although the blood–brain barrier is penetrable to serum proteins. On the other hand, patients with septic shock tend to have cytokines in both serum and CSF.


Mediators of Inflammation, 2, 23–25 (1993)

### Compartmentalization of TNF and IL-6 in meningitis and septic shock

Anders Waage,[1,2,CA] Alfred Halstensen,[3] Terje Espevik,[1] Petter Brandtzæg[4]

[1]Institute of Cancer Research and [2]Section of Haematology, University of Trondheim, and [3]Department of Internal Medicine, Haukeland University Hospital, Bergen, and [4]Department of Pediatrics, Ullevål University Hospital, Oslo, Norway

[CA] Corresponding Author

**We examined the compartmentalization of bioactive tumour necrosis factor (TNF) and interleukin 6 (IL-6) to the subarachnoid space and systemic circulation in patients with meningococcal meningitis and septic shock/bacteraemia. In patients with meningitis, median levels of TNF in 31 paired samples of cerebrospinal fluid (CSF) and serum were respectively 783 pg/ml and below detection limit ($p < 0.001$) and median levels of IL-6 were 150 ng/ml and 0.3 ng/ml ($p < 0.0001$). In patients with septic shock without meningitis, median levels in paired samples of CSF and serum were respectively below detection limit and 65 pg/ml (not significant, (ns)) (TNF, eleven patients) and 1.3 ng/ml–3 ng/ml (ns) (IL-6, nine patients). The data show that TNF and IL-6 are localized to the subarachnoid space in patients with meningitis although the blood–brain barrier is penetrable to serum proteins. On the other hand, patients with septic shock tend to have cytokines in both serum and CSF.**

**Key words:** Compartmentalization, IL-1, IL-6, Meningitis, Meningococcal disease, Septic shock, TNF

### Introduction

The blood–brain barrier is poorly penetrable to most water soluble substances except for a few nutrients and ions.[1] The barrier thus separates the subarachnoid space and the systemic circulation in two anatomically and functionally different compartments. However, during an inflammatory response the impenetrable character of the blood–brain barrier may be modified as evidenced by leakage of proteins from blood to CSF in meningitis.[2] The transport across the inflamed meninges is passive and directed by concentration gradients. In addition, hydrostatic forces favour transport from the circulation to the subarachnoid space. Although there is evidence for compartmentalization of the initial production of cytokines, little is known about how the altered permeability of the blood–brain barrier influences the distribution of cytokines in the organism.

The dominant clinical manifestations of meningococcal disease are meningitis and septic shock and the authors have previously studied the cytokine cascades separately in CSF and serum in this disease.[3,4] The dual presentation of the disease also provides an opportunity to study the function of the blood–brain barrier and the degree of compartmentalization of cytokines. In the present study this question is addressed by comparing the levels of TNF and IL-6 in paired samples of CSF and serum from the same patients. The results show that biologically active TNF and IL-6 are compartmentalized to the subarachnoid space in patients with meningitis, whereas the cytokines tend to be present in both compartments in patients with septic shock/bacteraemia.

### Patients and Methods

*Patients:* Characteristics of the patients and criteria of the diagnosis are described previously.[3,4] Samples were obtained from 32 male and 28 female patients. The age range was 2–93 years, 63% of the patients were 10–20 years of age. The patients had these manifestations: meningitis or meningitis and bacteraemia ($n = 33$), septic shock ($n = 9$), septic shock and meningitis ($n = 11$), and bacteraemia without meningitis or shock ($n = 7$). The patients were grouped in two categories, patients with meningitis, and patients with septic shock or bacteraemia. Patients with the combinations meningitis/septic shock or meningitis/bacteraemia were included in the first group as these patients have similar clinical courses and similar systemic concentrations of endotoxin.[5] Blood and CSF were drawn on admission prior to treatment with antibiotics. The samples were centrifuged, and frozen at −70°C.

*Assays for TNF, IL-1 and IL-6:* TNF levels were determined by the cytotoxic effect of TNF on the mouse fibrosarcoma cell line WEHI 164 clone 13 as described previously.[6] IL-6 was determined by the IL-6 dependent mouse hybridoma cell line B

© 1993 Rapid Communications of Oxford Ltd

13.29 clone 9 (kindly provided by Dr Aarden, University of Amsterdam) as described previously.[7]

*Statistics:* Differences between cytokine levels in paired samples were tested for significance with paired Wilcoxon rank sum test. $p$-values $<0.05$ in two-sided tests were considered to be significant.

### Results

Paired samples of CSF and serum were obtained from 31 patients with meningitis (Fig. 1A). Fourteen patients had no detectable TNF levels in either CSF or serum. In all positive samples, TNF was markedly higher in CSF (range 0–31070 pg/ml, median 783 pg/ml) than in serum (range 0–214 pg/ml, median 0), $p < 0.001$. Similarly, paired samples were obtained from eleven patients with bacteraemia/septic shock (Fig. 1B). In seven of these patients, TNF was higher in serum than in CSF, whereas two patients had higher levels in CSF than in serum (CSF, range 0–168 pg/ml, median 0. Serum, range 0–35712 pg/ml, median 65 pg/ml, ns difference).

*IL-6:* IL-6 is detected in a high proportion (86–100%) of both the CSF and serum samples.[3,4] Figure 2A demonstrates that 26 of 29 patients with meningitis from whom we obtained paired samples, had higher levels of IL-6 in CSF than in serum (CSF, range 0–859 ng/ml, median 150 ng/ml. Serum, range 0–164 ng/ml, median 0.3 ng/ml, $p < 0.0001$). In nine patients with septic shock/bacteraemia there was no significant difference between the IL-6 levels in the two compartments (CSF, range 0.1–807 ng/ml, median 1.3 ng/ml. Serum, range 0–2400 ng/ml, median 3 ng/ml, ns) (Fig. 2B).

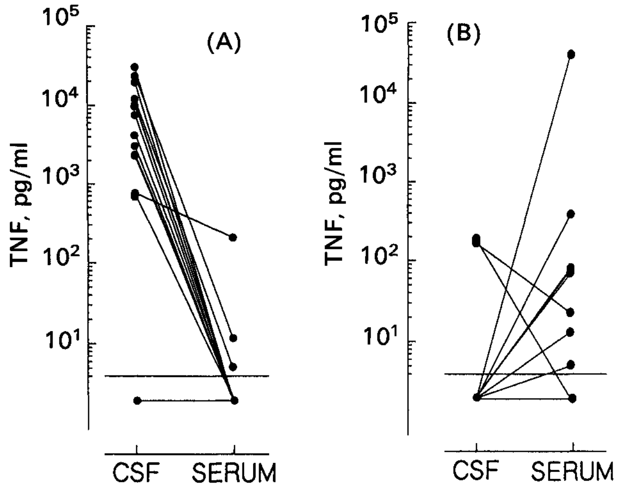

FIG. 1 (A) TNF levels in CSF and serum from 31 patients with meningitis. Each line denotes one patient, except the lowest one which denotes 14 patients with no detectable levels of TNF. (B) TNF levels in CSF and serum from eleven patients with septic shock/bacteraemia. The lowest denotes two patients with no detectable levels of TNF. Horizontal line indicates detection limit of the assay.

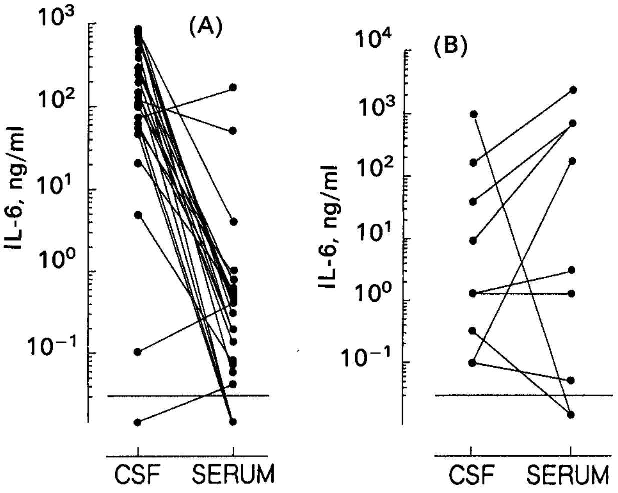

FIG. 2 (A) IL-6 levels in CSF and serum from 29 patients with meningitis. Each line denotes one patient. (B) IL-6 levels in CSF and serum from nine patients with septic shock/bacteraemia. Horizontal line indicates detection limit of the assay.

*Protein levels in CSF:* The protein concentration in CSF was analyzed because it reflects breakdown of the blood–brain barrier and the degree of communication between the subarachnoid space and systemic circulation. Patients with meningitis had median concentration of 3.7 g/l (range 0.2–13.5 g/ml) whereas patients with septic shock had 0.42 g/l (0.23–5.4 g/l), ($p < 0.5$). The data are in accordance with the current concept that serum proteins leak across the blood–brain barrier in meningitis.

### Discussion

The present study demonstrates that bioactive TNF and IL-6 are compartmentalized to the subarachnoid space in patients with meningococcal meningitis, whereas patients with septic shock have cytokines both in serum and CSF.

In patients with meningitis the blood–brain barrier becomes penetrable, as confirmed by elevated protein levels in CSF in meningitis patients in the present study. Others have suggested that TNF may leak from CSF to serum during meningitis.[8] However, in spite of the marked leakage of protein, and the high concentration gradient of TNF across the blood–brain barrier, the authors' data demonstrate that negligible amounts of bioactive TNF leak from CSF to serum during meningitis. By leakage from the subarachnoid space to the systemic circulation there is a dilution factor of 10–20. In addition, the cytokines may adhere to tissue and cells in the systemic circulation, and, furthermore, they may be inactivated by inhibitors and soluble receptors in serum. Apparently, these mechanisms are sufficient to minimize the effect of leakage of cytokines during meningitis.

In contrast, patients with septic shock/bacteraemia had a different pattern of cytokine presence. TNF and IL-6 were present in both serum and CSF and the ranges of concentrations overlapped. Apparently, there is no strict compartmentalization of bioactive TNF and IL-6 during septic shock/bacteraemia. A previous study has demonstrated that 50% of the patients with septic shock also have *Neisseria meningitidis* in CSF without elevated leukocyte counts.[5] The most reasonable interpretation of these observations is that bacteria are present and evoke a cytokine response also in the subarachnoid space in septic shock patients, although the host response does not include a classical inflammatory reaction with elevated leukocyte counts at the time of sampling. An alternative interpretation that cytokines leak across the blood–brain barrier is not likely because a comparison between serum proteins in serum and CSF indicate negligible leakage. From a pathophysiological point of view compartmentalization of cytokines may help to localize the inflammatory reaction during meningitis.

### References

1. Bradbury MWB. The structure and function of the blood–brain barrier. *Fed Proc* 1984; **43:** 186–190.
2. Quagliarello VJ, Ma A, Stukenbrok H, Palade GE. Ultrastructural localization of albumin transport across the cerebral microvasculature during experimental meningitis in rat. *J Exp Med* 1991; **174:** 657–672.
3. Waage A, Halstensen A, Shalaby R, Brandtzaeg P, Kierulf P, Espevik T. Local production of tumor necrosis factor a, interleukin 1, and interleukin 6 in meningococcal meningitis. Relation to the inflammatory response. *J Exp Med* 1989; **170:** 1859–1867.
4. Waage A, Espevik T, Halstensen A. Association between tumour necrosis factor in serum and fatal outcome in patients with meningococcal disease. *Lancet* 1987; **i:** 355–357.
5. Brandtzaeg P, Kierulf P, Gaustad P, *et al.* Plasma endotoxin as a predictor of multiple organ failure and death in systemic meningococcal disease. *J Infect Dis* 1989; **159:** 195–204.
6. Espevik T, Nissen-Meyer J. A highly sensitive cell line, WEHI 164 clone 13, for measuring cytotoxic factor/tumor necrosis factor from human monocytes. *J Immunol Methods* 1986; **95:** 99–105.
7. Aarden LA, Landsdorp PM, de Groot E. A growth factor B cell hybridomas produced by human monocytes. *Lymphokines* 1985; **10:** 175–185.
8. Møller B, Mogensen SC, Wendelboe P, Bendtzen K, Munck Petersen C. Bioactive and inactive forms of tumor necrosis factor-α in spinal fluid from patients with meningitis. *J Infect Dis* 1991; **163:** 886–889.

ACKNOWLEDGEMENTS. The study was carried out with grant support from The Norwegian Cancer Society and the Norwegian Council for Science and Humanities. We thank Berit Størdal and Mari Sørensen for technical assistance, and Dagmar Moholdt for typing the manuscript.

**Received 16 September 1992; accepted 20 October 1992**
